# Interaction between early *in ovo* stimulation of the gut microbiota and chicken host – splenic changes in gene expression and methylation

**DOI:** 10.1186/s40104-021-00602-1

**Published:** 2021-07-07

**Authors:** A. Dunislawska, A. Slawinska, M. Gryzinska, M. Siwek

**Affiliations:** 1 Department of Animal Biotechnology and Genetics, UTP University of Science and Technology, 85-084 Bydgoszcz, Poland; 2 Institute of Biological Basis of Animal Production, Sub-Department of General and Molecular Genetics, University of Life Sciences in Lublin, 20-032 Lublin, Poland

**Keywords:** Gene expression, Host-microbiome immune tissue, Interaction, Methylation level

## Abstract

**Background:**

Epigenetic regulation of the gene expression results from interaction between the external environment and transcription of the genetic information encoded in DNA. Methylated CpG regions within the gene promoters lead to silencing of the gene expression in most cases. Factors contributing to epigenetic regulation include intestinal microbiota, which in chicken can be potently modified by *in ovo* stimulation. The main aim of this study was to determine global and specific methylation patterns of the spleen under the influence of host-microbiome interaction.

**Results:**

Fertilized eggs of two genotypes: Ross 308 and Green-legged Partridgelike were *in ovo* stimulated on d 12 of incubation. The injected compounds were as follows: probiotic – *Lactococcus lactis* subsp*. cremoris* IBB477, prebiotic – galactooligosaccharides, and synbiotic – combination of both. Chickens were sacrificed on d 42 post-hatching. Spleen was collected, RNA and DNA were isolated and intended to gene expression, gene methylation and global methylation analysis. We have proved that negative regulation of gene expression after administration of bioactive substances *in ovo* might have epigenetic character. Epigenetic changes depend on the genotype and the substance administered *in ovo*.

**Conclusion:**

Epigenetic nature of microbial reprogramming in poultry and extension of issues related to host-microbiome interaction is a new direction of this research.

## Background

Epigenetic regulation of the gene expression reflects in particular the interaction of the external environment with the genetic information. There are potentially heritable changes in gene expression which do not involve alteration in DNA sequence. Changes in the gene expression may be triggered by factors such as: nutrition, health, climate or stress [1]. Epigenetic mechanisms responding to these environmental factors include: expression of microRNA (miRNA) sequences, histone modification and DNA methylation. The methylation process might be influenced by many components present in the chicken diet such as probiotic bacteria [2] and also, the prebiotic fermentation product: the short-chain fatty acid, called butyrate [3, 4]. These components mainly modulate the microbiota of the gastrointestinal tract (GIT), which affects the host organism by regulating its immune responses, metabolism, digestive processes, or nutrient absorption [5]. Supplementation by bioactive substances can directly modulate composition of the host microbiota, which indirectly acts on the entire host organism [6]. Modulation effects depend on the time and mode of administration of the bioactive substance [7]. In chickens prebiotics, probiotics or synbiotics are routinely delivered in-feed and in-water directly after hatching [6]. Supplementation with bioactive substances is continued for another 2 weeks [6]. During this period GIT is colonized by beneficial bacteria [8]. In poultry, an alternative route of bioactive substances administration is provided by *in ovo* technology. It is based on a single dose of prebiotics, probiotics or synbiotics injected on the d 12 of embryonic development into the air chamber of the egg [9–12]. Such a route of delivery ensures contact of GIT with bioactive substances as early as possible. In the previous studies we proved efficiency of *in ovo* technology for reprogramming the chicken microbiome at an early stage of embryonic development by administering prebiotics (e.g. inulin, GOS, RFO) or synbiotics (e.g. inulin + *Lactococcus lactis* subsp. *lactis* IBB2955, GOS + *Lactobacillus salivarius* IBB3154, RFO + *Lactobacillus plantarum* IBB3036) [10–12].

DNA methylation can influence gene expression [13]. DNA methylation also is characterized by the stability of cytosine modifications within the CpG dinucleotides [13]. Remodeling of DNA methylation in genome can be global or locus-specific [14]. As showed by studies performed on monkeys, during embryogenesis, global demethylation of DNA occurs, and a de novo methylation profile appears at the blastocyst stage [15]. Therefore, the chicken environment, egg composition and conditions of fertilized eggs incubation might significantly influence an embryo DNA methylation. DNA requires donors of methyl groups and cofactors, originating from the outside environment (e.g. feed). Studies have repeatedly confirmed DNA methylation as a conservative epigenetic mechanism in organism development [16, 17]. However, little is still known about tissue-specific DNA methylation patterns in chicken.

It has been generally recognized that the overall pattern of the DNA methylation is conserved in many species [18, 19]. The knowledge about these patterns and the epigenetic regulation of gene expression in poultry is still scarce. We hypothesize that in chickens stimulated with bioactive substances *in ovo* on the d 12 of embryonic development, an epigenetic mechanism regulating gene expression is developed. Therefore, the main aim of this study was to determine global and specific methylation patterns of the main immune organ as spleen under the influence of host-microbiome interaction. We carried out this analysis in a few steps: (1) meta-analysis of the whole transcriptome data of immune organs, to select candidate genes for DNA methylation analysis; (2) estimation of the global methylation level in spleen stimulated *in ovo*; (3) estimation of the expression and CpG methylation level of candidate genes in spleen. The innovativeness of the study is expressed by acquiring new knowledge about the mechanisms of gene silencing, where the gene expression regulation resulted from the stimulation of chicken microbiome by bioactive substances injected *in ovo*.

## Methods

### Experimental outline

In the first stage of the study, (1) a meta-analysis was performed on the basis of whole-transcriptome microarray data generated in previous experiments [10, 12]. This step was crucial in order to select immune-related genes that were silenced. Four bioactive compounds were used in those experiments; two prebiotics (P1 – galactooligosaccharides (GOS); P2 – inulin) and two synbiotics (S1 – *Lactococcus lactis* subsp. *lactis* IBB2955 + inulin; S2 – *Lactobacillus salivarius* IBB3154 + GOS, S3 – *Lactococcus lactis* subsp. *cremoris* IBB477 + GOS).

The present experiment follows the same route of the substance delivery and focuses on the synbiotic and its individual components: prebiotic (GOS) and probiotic (*Lactococcus lactis* subsp. *cremoris* IBB477). These three bioactive substances (prebiotic, probiotic, synbiotic) were *in ovo* administered into chicken embryos of two contrasting chicken genotypes: Ross 308 and Green-legged partridge.

Based on the spleen isolated from *in ovo* stimulated chickens following analyzes were performed: (2) global methylation to verify the epigenetic nature of the changes, and then (3) gene expression analysis (to confirm expression regulation under the influence of various substances) and (4) methylation analysis of individual selected genes.

### Meta-analysis of published microarray data for gene selection

Gene selection for methylation analysis was based on two sets of microarray data. These data sets contained broiler chicken transcripts generated from individuals which received prebiotic and synbiotic *in ovo* on d 12 of egg incubation [10, 12]. Both projects carried out whole-genome microarray analyzes (Affymetrix, Santa Clara, US), based on genetic material isolated from spleen in experiment with *in ovo* injection of S1, S2 and S3. Meta-analysis aimed to select genes which were silenced at mRNA level in spleen. The analysis was carried out based on gene lists generated by Affymetrix Expression Console software. In silico selection of gene sequences followed these criteria: *P*-value (*P* < 0.05) and fold change (down-regulation; FC < − 1.0). Subsequently, selected gene groups were compared with each other based on the Venn diagrams. Connection of the selected genes was analyzed with STRING [20]. A final list was selected based on gene function and possibility of designing primers for the RT-qPCR and qMSP reaction.

### Experimental setup and tissue collection

Six hundred eggs of Ross 308 (Ross) broiler chicken and 600 eggs of Green-legged Partridge (GP) were incubated in a commercial hatchery using an automated incubator at 37.8 °C and a relative humidity of 61–63%. On the d 12 of incubation, eggs were randomly distributed into three experimental groups (150 eggs per group): (1) probiotic (PRO) – *Lactococcus lactis* subsp. *cremoris* IBB477, (2) prebiotic (PRE) – GOS (Bi2tos; Clasado Biosciences, Ltd., Jersey, UK) (3) synbiotic (SYN) – *Lactococcus lactis* subsp. *cremoris* IBB477 with GOS. The set concentration of bacteria was 10^5^ bacteria CFU/egg and the concentration of prebiotic was 3.5 mg/egg (dose optimization described in Dunislawska et al. 2017 [11] and Bednarczyk et al. 2016 [21]). The control group (C) was mock-injected with 0.2 mmol/L physiological saline (0.9%). Eggs were injected into an air cell with 0.2 mL of aqueous solution of each substance. After hatching, birds were housed in litter pens (4 replicates/group, 8 animals each). Feed and water was delivered manually and was available ad libitum. The temperature was regulated with infrared bulbs according to the age of the birds and the natural light was available. Feeding regime was applied according to the requirements of the given genotype. Table 1 presents the chemical composition of the feed offered to broilers and native chickens. Eight randomly selected individuals from each group (PRO, PRE, SYN and C) were sacrificed on the d 42 post-hatching and spleen was collected.

**Table 1:** Chemical composition of commercial feeds used for broilers and native chickens

Items	Broilers				Green-legged Partridge	
	Starter (d 1–10)	Grower I (d 11–21)	Grower II (d 22–33)	Finisher (d 34–42)	Starter (d 1–28)	Grower (d 19–42)
ME_N_, MJ/kg	12.50	12.95	13.35	13.41	11.9	11.7
Crude protein, g/kg	220	200	190	184	200	185
Crude fiber, g/kg	28.0	30.0	31.0	32.0	34.0	35.0
Lysine, g/kg	13.8	12.5	11.3	10.5	11.0	10.0
Methionine+Cystine, g/kg	10.3	9.5	8.8	8.2	8.2	7.2
Threonine, g/kg	9.2	8.3	7.6	7.2	7.6	7.0
Tryptophan, g/kg	2.2	2.0	1.9	1.9	2.1	2.0

### RNA and DNA isolation

Tissues for RNA isolation were fixed in stabilizing buffer (fix RNA, EURx, Gdansk, Poland). RNA isolation was prepared by using TRI reagent (MRC, Cincinnati, USA) and commercial kit for RNA purification (Universal RNA Purification Kit, EURx, Gdansk, Poland). Spleen (*n* = 6/each group) was homogenized with the TissueRuptor homogenizer (Qiagen GmbH, Hilden, Germany) in TRI reagent. Isolation of DNA from spleen was carried out by phenol – chloroform method [22] as described by Dunislawska et al. 2020 [23]. RNA and DNA quality and quantity was checked by electrophoresis and NanoDrop2000 (Scientific Nanodrop Products, Wilmington, USA).

### Global methylation analysis

Global DNA methylation analysis was prepared using commercial set for methylated DNA quantification (MDQ1, Imprint Methylated DNA Quantification Kit, Sigma-Aldrich) according to manufacturer’s protocol and based on Gryzinska et al. 2013 [24]. DNA isolated spleen (*n* = 6/each group) was diluted in binding solution to final concentration 150 ng/μL. DNA was intended for estimation of methylated DNA level based on the ELISA principle on 96-well plates. Positive (methylated) and blank controls were analyzed together with DNA samples. The absorbance was measured at 450 nm. For each stimulated group, six samples, each derived from a different individual, were analyzed. The absorbance measurements were performed in two technical repeats, each using the same amount of DNA. Two measurements were averaged, and the mean value was used for the further analyses. Global DNA methylation levels are percentages relative to the methylated control and were calculated using the following formula: $\frac{A_{450}S-{A}_{450}B}{A_{450} MC-{A}_{450}B}\ x\ 100\%$, where *A*_450_*S* is the average absorbance of the sample, *A*_450_*B* is the average absorbance of the blank, *A*_450_*MC* is the average absorbance of the methylated control. Statistical analysis was carried out by SAS Enterprise Guide 8.2 (SAS Institute Inc., Cary, NC, USA). The quantitative values (percentage of global methylation) were first analyzed for normality using the Shapiro – Wilk test. The homogeneity of variance test (Levene’s test) was also carried out. The obtained results met the assumptions about the normal distribution and homogeneity of variance, the two-way ANOVA and Duncan test were used (*P* < 0.05).

### Gene expression analysis – RT-qPCR

Gene expression analysis was performed by quantitative reverse transcription PCR (RT-qPCR). cDNA was synthesized using Maxima First Strand cDNA Synthesis Kit for RT-qPCR (Thermo Scientific/Fermentas, Vilnius, Lithuania), following the manufacturer’s recommendations. The qPCR reaction mixture included Maxima SYBR Green qPCR Master Mix (Thermo Scientific/Fermentas, Vilnius, Lithuania), 1 μmol/L of each primer and diluted cDNA (140 ng). Thermal cycling was performed in a LightCycler II 480 (Roche Diagnostics, Basel, Switzerland). Each RT-qPCR reaction was conducted in two technical replicates and in 24 biological repeats (*n* = 6/each group). Gene expression analysis was performed for selected genes in meta-analysis step. Sequences of primers were based on the literature or were designed by NCBI Primer BLAST tool [25] based on NCBI sequence. Primer sequences are shown in Table 2. Relative gene expression analysis was conducted separately for each experimental group by the ΔΔCt method [26] using *ACTB* [27] and *G6PDH* [28] as reference genes. Geometric means of cycle threshold (Ct) values of reference genes were used in the analysis [29]. Statistical analysis was carried out by SAS Enterprise Guide 8.2 (SAS Institute Inc., Cary, NC, USA). The quantitative values (delta-Ct values) were first analyzed for normality (Shapiro – Wilk test) and and homogeneity of variance (Levene’s test). The obtained results of most genes met the assumptions about the normal distribution. For genes where the distribution was not normal, the data was transformed to lead to the assumption that the distribution was normal. A one-way ANOVA and Duncan test was performed (effect of substances within a given genotype; *P* < 0.05).

**Table 2:** Primer sequences used in the RT-qPCR reaction

Gene	Primers sequence (5'→3')	Amplicon size, bp	NCBI no.
*CD72 (chB1)*	F: AGGAAGGTAGGGCAGCAATG R: CTGACCTGAGGTTCGCCAAA	134	395923
*CXCR5*	F: GCTCTGACTGTAGGGTGACG R: TGAAATGATGGGCAGTGGCT	145	419784
*NFATC1*	F: TCGAGTTCAAGCACAGCGAT R: GAAGGACCCCCTCGGAAGA	155	420815
*SYK*	F: AAGGGACAGCAATGGTTCCT R: AATTTAACAGACCTGCCAGAGG	142	427272
*CYR61*	F: ATCGCTCGTTCAGACGCATA R: TGTCTGGGCTCCGCTAAAAG	144	429089
*NR4A3*	F: GGCATCCCCGGAGTTTCTCTG R: TTTGACGAGGCCGCTCATT	237	420996
*SERPING1*	F: GTCCTCGTGCCACACTTACC R: TTGACCAATGCTTGCCCACC	111	423132
*TNFRSF14*	F: TGAGCACCATCAGGGGTATC R: AGGTACGGATGCTTCCCAAG	170	420403
*IKZF1*	F: GCGTGTGAAAGAGCGACTTC R: GAACACTCCGCACAACACCT	149	395974
*KLHL6*	F: ATGGTTTCTGCGTCAACTCC R: CATCCTGGCTGGGATGCAATA	120	424762
*ANGPTL4*	F: TCCTCGATTCGCGAGTTCTG R: CAGGGCACTGGGAGCTG	148	769087

### *Gene methylation analysis-* qMSP

DNA methylation is a specific chemical modification of a nucleic acid, involving the attachment of a methylene group to cytosine or adenine nucleotides. The isolated DNA was subjected to methylation analysis using the qMSP method as described by Dunislawska et al. 2020 [23]. The qMSP method is a methyl specific quantitative PCR (qPCR) preceded by DNA conversion using bisulfite. Due to this modification, unmethylated cytosine undergoes deamination and uracil is formed. In contrast, 5-methylcytosine (which is a methylation product) is resistant to this modification, making it possible to make it visible in the qPCR reaction with the help of primers specific for methylated and unmethylated DNA. The mechanism of methylation concerns only cytosines that are part of the cytidine-phosphate-guanosine (CpG) dinucleotide sequence. Therefore the primers for qMSP reactions were designed within CpG islands. Based on the selected gene list in meta-analysis, gene primers in two variants were designed: methylated and unmethylated (Table 3). The conversion was carried out using the EpiJet Bisulfite Conversion Kit (Thermo Fisher Scientific/Fermentas, Vilnius, Lithuania) according to the manufacturer’s instructions. In the next stage, the qPCR reaction was performed for the selected genes, where for each gene two primer pairs were designed - specific for methylated and non-methylated DNA using the MethPrimer tool. Primers for qMSP were complementary to the gene promoter region and were designed based on criteria: island size > 100, GC% > 50.0; obs./exp. > 0.60. DNA oligonucleotides were synthesized by Sigma-Aldrich. The qPCR analysis was performed in LightCycler 480 (Roche Diagnostics, Risch-Rothreuz, Switzerland) thermal cycler. The reaction mixture contained the Maxima SYBR Green qPCR Master Mix intercalating dye (Thermo Fisher Scientific/Fermentas, Vilnius, Lithuania). The optimized melting temperature was 58 °C. After amplification, a melting curve was generated for each product (*n* = 6/group). This was due to a gradual increase in temperature up to 98 °C with continuous measurement of fluorescence. The relative level of DNA methylation (%) was calculated based on the results of melting curves (read fluorescence level) for each individual according to the formula [30]: $\% of\ methylation=100\ x\left(\ \frac{M}{M+U}\right),$ where M - average fluorescence intensity of the methylated product, U - average fluorescence intensity of the unmethylated product. Statistical analysis was carried out by SAS Enterprise Guide 8.2 (SAS Institute Inc., Cary, NC, USA). The quantitative values (percentage of methylation) were first analyzed for normality (Shapiro – Wilk test) and homogeneity of variance (Levene’s test). The obtained results of most genes met the assumptions about the normal distribution. For genes where the distribution was not normal, the data was transformed to lead to the assumption that the distribution was normal. A one-way ANOVA and Duncan test was performed (effect of substances within a given genotype; *P* < 0.05).

**Table 3:** Sequences of the primers designed to qMSP reaction by using MethPrimer tool

Gene		Primers sequence (5'→3')	GC%	Amplicon size, bp	NCBI no.
*CD72*	M	F: AACGGGTTATGTGTCGTTATTAGTC R: AAACTAAACCCTACTACCTTCTCGC	60.00 72.00	107	395923
	U	F: TGGGTTATGTGTTGTTATTAGTTGT R: ACTAAACCCTACTACCTTCTCACA	64.00 70.83	103	
*CXCR5*	M	F: AGAGGTTGGGATTTACGGTAATAAC R: ACAACTTTCTACCTTTACAAACGCT	56.00 56.00	156	419784
	U	F: AGGTTGGGATTTATGGTAATAATGT R: ACAACTTTCTACCTTTACAAACACT	56.00 56.00	154	
*NFATC1*	M	F: CGATTCGGAAATATTAATAAAGC R: AAAAATAATATAAACCCTACCCGAC	52.17 60.00	100	420815
	U	F: TTTGTATTGATTTGGAAATATTAATAAAGT R: ACAAAAATAATATAAACCCTACCCAAC	46.67 62.96	109	
*SYK*	M	F: TATTAGGCGTTTTCGGGAAC R: AAATTAATACATTTACTCGCCGCT	70.00 54.17	115	427272
	U	F: GTTTATTAGGTGTTTTTGGGAATGA R: CCAAATTAATACATTTACTCACCACT	68.00 57.69	120	
*CYR61*	M	F: TTTGGTTTTAGTGTTTAAAGACGT R: TTATATTTACCTTCAAAAAAACGTA	58.33 44.00	150	429089
	U	F: TTTTGGTTTTAGTGTTTAAAGATGT R: TATTTATATTTACCTTCAAAAAAACATA	56.00 42.86	154	
*NR4A3*	M	F: GGGAAAGGATAAAGTTTTTGTAGTC R: AAACTCAAACGTAACCCTAAACGTA	52.00 56.00	179	420996
	U	F: GGGAAAGGATAAAGTTTTTGTAGTTG R: AAACTCAAACATAACCCTAAACATA	53.85 56.00	179	
*SERPING1*	M	F: GGTAACGAGAGTTTGGATTTGTAAC R: CCTAAATAAACCCTAAAAACTACGC	56.00 64.00	163	423132
	U	F: TGGTAATGAGAGTTTGGATTTGTAAT R: CTAAATAAACCCTAAAAACTACACC	53.85 64.00	163	
*TNFRSF14*	M	F: GTTTTAGTTATTTTTGTTTTTACGTTCGT R: CCGCTATCACTATACAACTTCTCG	65.52 62.50	298	420403
	U	F: AGTTATTTTTGTTTTTATGTTTGT R: CACTATCACTATACAACTTCTCACC	62.50 64.00	292	
*IKZF1*	M	F: GTAGTAGTAATTGTTGGAGGAGGC R: AAAAATAACTTTACGAAACAACGAA	62.50 64.00	192	395974
	U	F: GTAGTAGTAATTGTTGGAGGAGGTG R: AAAAATAACTTTACAAAACAACAAA	64.00 64.00	192	
*KLHL6*	M	F: TTTTTTGGATAATGAGTGTTTAACG R: AAACACCAAAAAAAATCCCGTA	52.00 63.64	100	424762
	U	F: TTTTTGGATAATGAGTGTTTAATGA R: CTAAAACACCAAAAAAAATCCCATA	48.00 64.00	102	
*ANGPTL4*	M	F: TAATTTTAACGGGAAGTATTTTCGT R: CAACTTTAAAACTCTACCTCCAACG	56.00 60.00	156	769087
	U	F: TAATTTTAATGGGAAGTATTTTTGT R: ACTTTAAAACTCTACCTCCAACACA	56.00 60.00	154	

*M* specific for methylated DNA, *U* specific for unmethylated DNA

Interaction analysis for both gene expression and gene methylation was also performed. The significance of effects: genotype, substance and interaction genotype x substance were calculated with two-way ANOVA (SAS Enterprise Guide 8.2 update 4; SAS Institute Inc., Cary, NC, USA).

## Results

### Meta-analysis based on published microarray data and gene selection

**Fig. 1:**
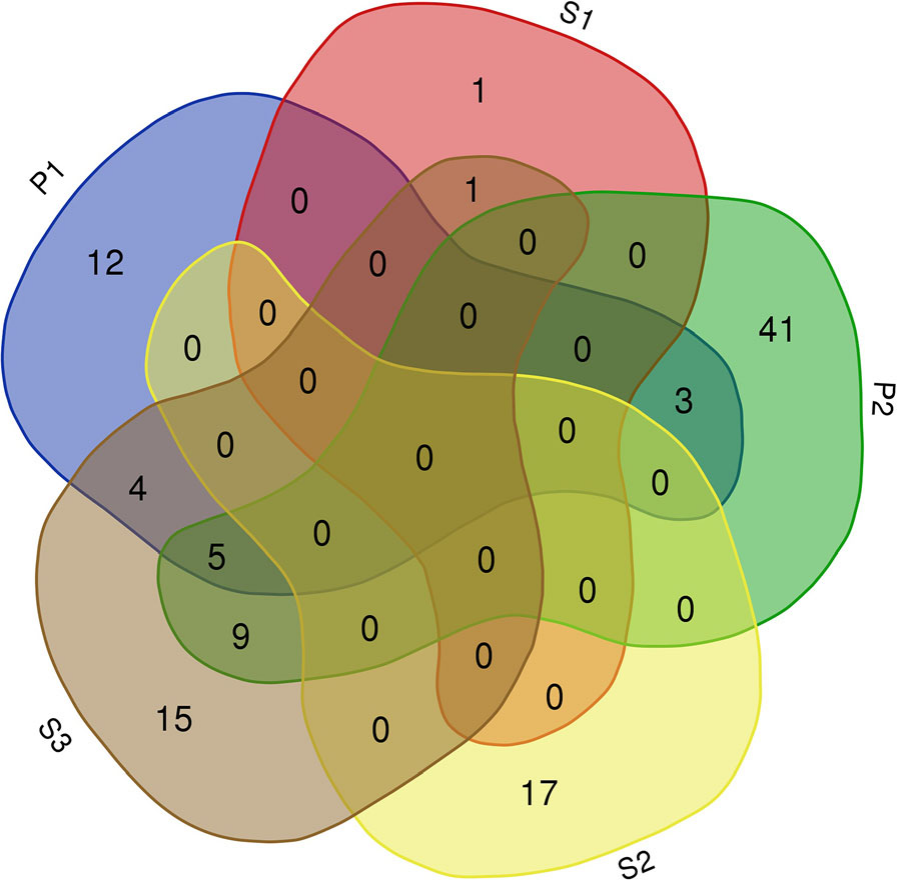
Venn diagram. The number of down-regulated genes in spleen detected in five experimental groups injected *in ovo* with: P1 – GOS; P2 – inulin; S1 – *Lactococcus lactis* subsp. *lactis* + inulin; S2 – *Lactobacillus salivarius* + GOS, S3 – *Lactococcus lactis* subsp. *cremoris* + GOS

The meta-analysis of high-throughput transcriptome data (expression microarrays) allowed the identification of down – regulated genes in spleen. The gene expression was silenced after synbiotics and prebiotics administration *in ovo*. The comparison of down regulated genes is presented in Venn diagram (Fig. 1).

**Fig. 2:**
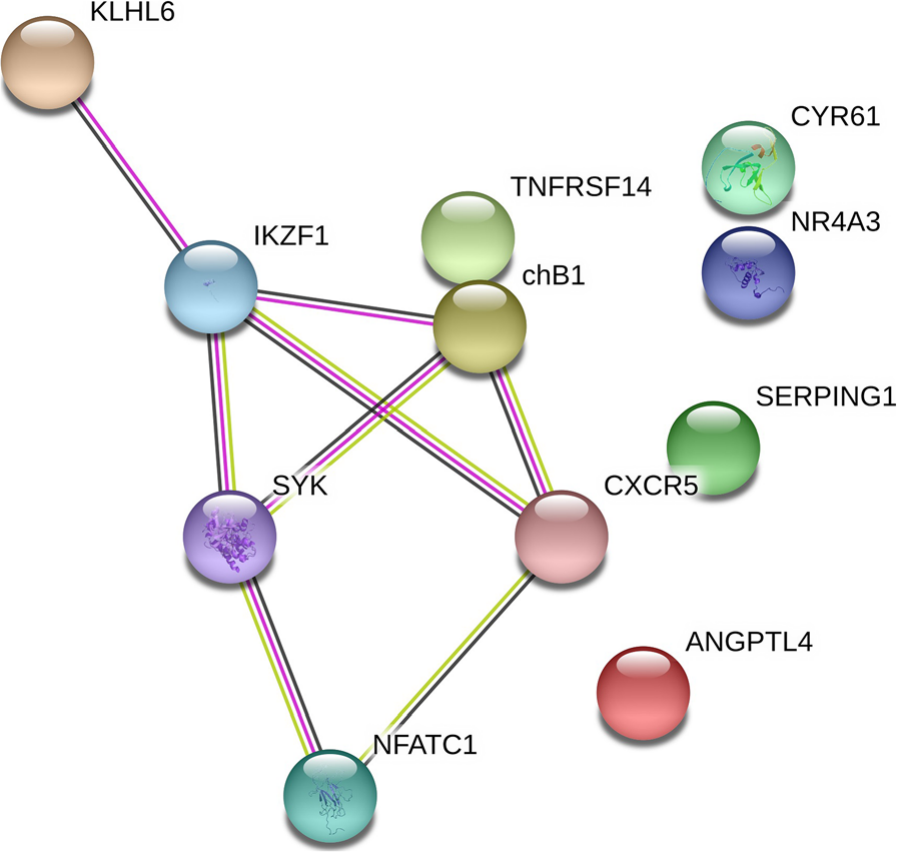
Analysis of the relationship between proteins encoded by down-regulated genes selected based on microarray data

Analysis showed 108 down-regulated genes in spleen for five analyzed experimental groups. The selected gene sequences and their connection are presented in Fig. 2. A connection was shown between 6 genes. These gene were intended for further analysis using the qMSP reaction.

### Global methylation

The results of global methylation of spleen after *in ovo* stimulation with prebiotic, probiotic and synbiotic *in ovo* are presented in the Table 4. There are statistically significant differences between the analyzed genotypes (GP and Ross; *P* < 0.0001) and between the probiotic and prebiotic/synbiotic injected groups in Ross genotype (*P* = 0.0003).

**Table 4:** The mean global (total; percentage) level of methylation of cytosine in DNA in the spleen

Genetic group	Substance	Mean	SD	CV
Ross	C	11.79^a^	6.41	54.33
	PRE	29.74^b^	24.55	82.53
	PRO	6.82^a^	1.18	17.35
	SYN	28.67^b^	15.79	55.08
GP	C	10.56	4.32	40.88
	PRE	9.79	2.48	25.27
	PRO	8.05	2.05	25.44
	SYN	12.31	5.76	46.84

*SD* standard deviation, *CV* coefficient of variation; *P* < 0.05; a,b – differences between groups

### Relative gene expression

**Fig. 3:**
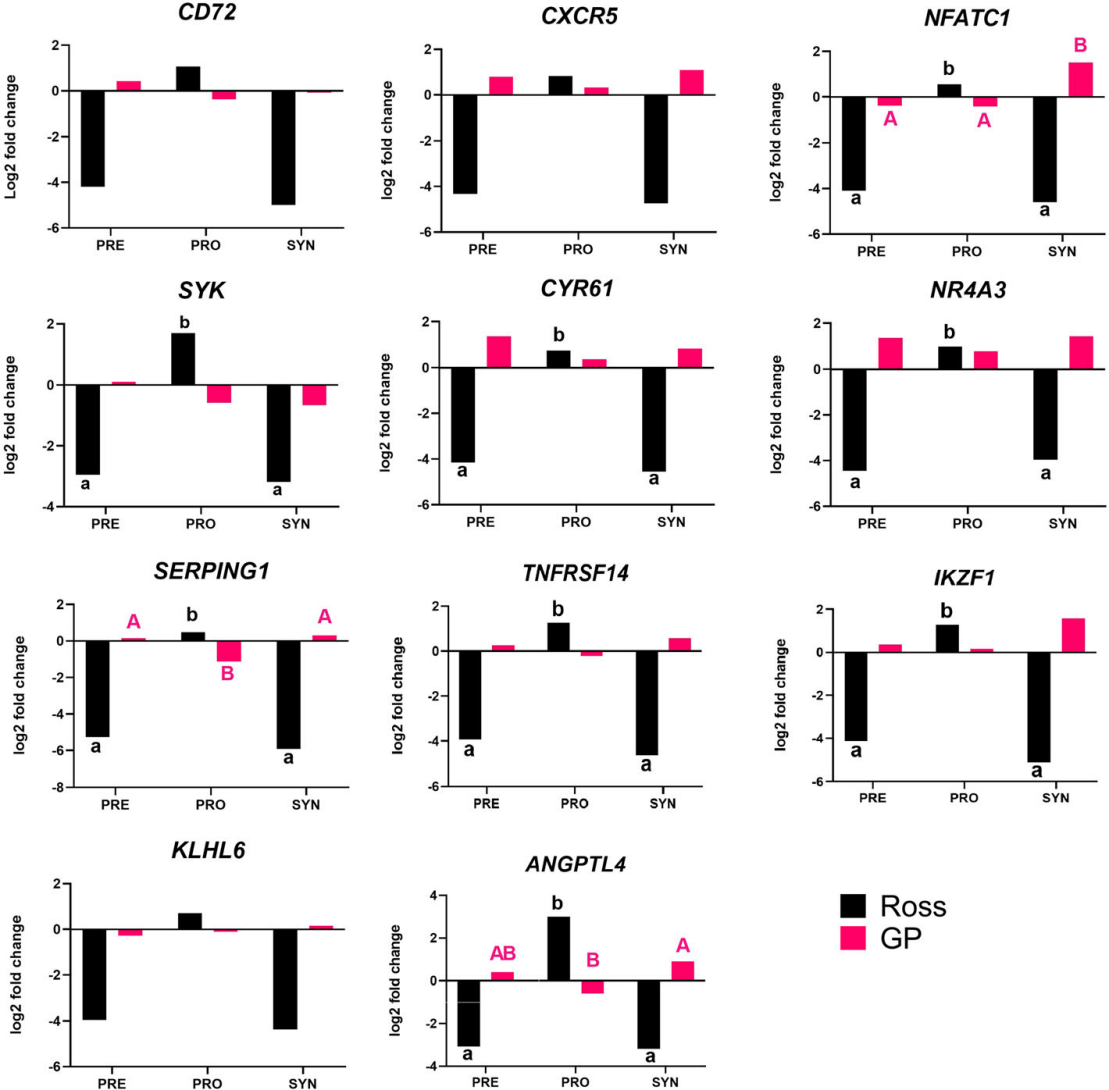
Changes in relative expression of the selected gene panel in spleen of Ross and GP injected *in ovo* with PRE - prebiotic, PRO - probiotic and SYN - synbiotic (mRNA abundance – LOG_2_ Fold Change) *P* < 0.05; A,B,C – comparison between groups in GP, a, b- comparison between groups in Ross (based on one-way ANOVA and Duncan test)

Significant changes in the expression of 8 genes were found: *NFATC1* (*P* < 0.0001), *SYK* (*P* = 0.0042), *CYR61* (*P* < 0.0001), *NR4A3* (*P* < 0.0001), *SERPING1* (*P* < 0.0001), *TNFRSF14* (*P* < 0.001), *IKZF1* (*P* < 0.0001) and *ANGPTL4* (*P* < 0.0001) in Ross, and also 3 genes in GP: *NFATC1* (*P* = 0.0482), *SERPING1* (*P* = 0.0015) and *ANGPTL4* (*P* = 0.0346). Gene expression analysis showed a statistically significant decrease in the expression of 8 selected genes after the administration of a prebiotic and a synbiotic and increase of expression of these genes after probiotic administration in Ross (*P* < 0.05) There was a significant increase in: *NFATC1, SERPING1* and *ANGPTL4* expression after administration of the synbiotic and also *SERPING1* and *ANGPTL4* after prebiotic in GP. The administration of the probiotic significantly decreased expression of these genes. The results are presented in the Fig. 3.

### Gene methylation analysis

**Fig. 4:**
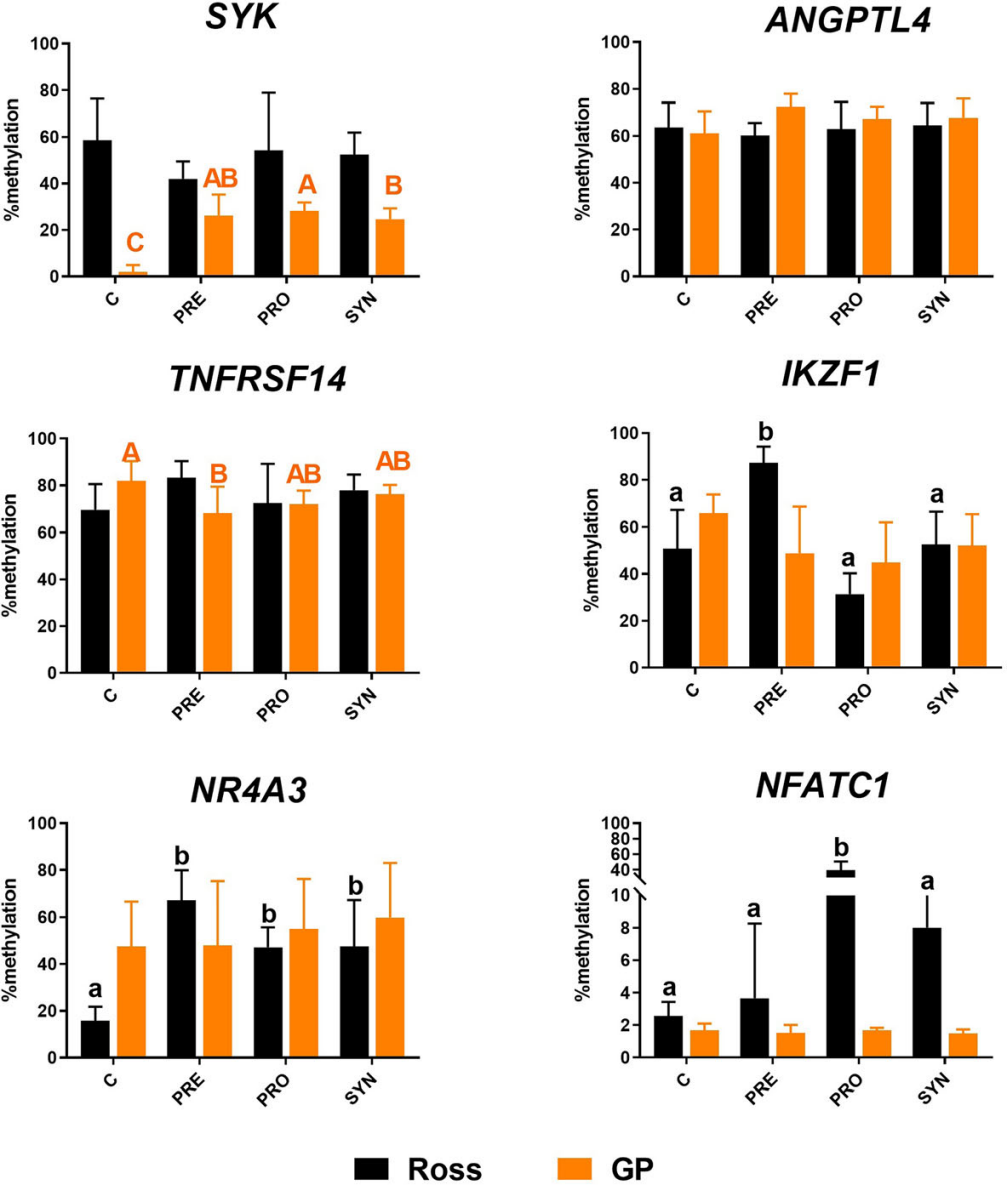
DNA methylation of the *SYK, ANGPTL4, TNFRSF14, IKZF1, NR4A3, NFATC1* genes in spleen. X-axis – genetic groups: Ross and Green-legged Partridge (GP); groups: C – control, PRO – probiotic, PRE – prebiotic, SYN – synbiotic. Y-axis – percentage of methylation; *n* = 6/each group. *P* < 0.05; A,B,C – comparison between groups in GP, a, b- comparison between groups in Ross (based on one-way ANOVA and Duncan test). SD values are also marked in the graph (n = 6/each group)

DNA methylation analysis shows statistically significant changes (*P* < 0.05) in the level of methylation among substances within a genotype. The differences were detected for 5 genes: *SYK (P* < 0.0001)*, TNFRSF14 (P* = 0.0406) in GP and *IKZF1 (P =* 0.0024)*, NR4A3 (P* = 0.008) and *NFATC1 (P =* 0.0043) in Ross. A statistically significant increase in the methylation level in *SYK* gene equals: 2% in C group, 24% in SYN, 26% in PRE and 28% in PRO group in GP genotype. The methylation level of *TNFRSF14* gene in GP genotype decreased from 81% in C group to 72% in PRO and 68% in PRE group. *IKZF1* methylation decreases in Ross after PRO administration by approximately 20%. However, after administration of the PRE in Ross, *IKZF1* methylation level increases from 50% to 87%. The administration of bioactive substances significantly increased the methylation of the *NR4A3* from 15% in the C group in the spleen of Ross, to 47% in PRO and SYN groups and 67% in PRE group. Methylation of *NFATC1* increased from 2% in C group to 39% in PRO group. The results are presented in the Fig. 4.

Analysis performed with two-way ANOVA (*P* < 0.05) showed influence of genotype on expression of 6 genes (*NFATC1, SYK, SERPING1, IKZF1, KLHL6 and ANGPTL4)* and methylation of 3 genes (*NFATC1, SYK* and *KLHL6*) and also the influence of substance on expression of 7 genes (*NFATC1, CYR61, NR4A3, SERPING1, TNFRSF14, IKZF1, ANGPTL4)* and methylation of 2 genes (*NFATC1* and *IKZF1*). A genotype x substance interaction analysis showed influence on expression of 7 genes (*NFATC1, CYR61, NR4A3, SERPING1, TNFRSF14, IKZF1* and *ANGPTL4*) and methylation of 4 genes (*NFATC1, SYK, TNFRSF14, IKZF1)* (Table 5).

**Table 5:** Effects of genotype and substance delivered *in ovo*, and their interaction on gene expression and methylation signatures in spleen of Ross and GP chickens

Gene	Gene expression			Gene methylation		
	Genotype	Substance	Genotype × Substance	Genotype	Substance	Genotype × Substance
*CD72*	ns	ns	ns	ns	ns	ns
*CXCR5*	ns	ns	ns	ns	ns	ns
*NFATC1*	< 0.05	< 0.0001	< 0.0001	< 0.0001	< 0.01	< 0.01
*SYK*	< 0.01	ns	ns	< 0.0001	ns	< 0.01
*CYR61*	ns	< 0.0001	< 0.0001	ns	ns	ns
*NR4A3*	ns	< 0.0001	< 0.0001	ns	ns	ns
*SERPING1*	< 0.001	< 0.0001	< 0.0001	ns	ns	ns
*TNFRSF14*	ns	< 0.0001	< 0.0001	ns	ns	< 0.05
*IKZF1*	< 0.0001	< 0.0001	< 0.0001	ns	< 0.01	< 0.01
*KLHL6*	< 0.05	ns	ns	< 0.0001	ns	ns
*ANGPTL4*	< 0.0001	< 0.001	< 0.0001	ns	ns	ns

Significance levels: *P* < 0.05; *P* < 0.01; *P* < 0.001; *P* < 0.0001 and *P* > 0.05 (non-significant, ns)

Gene expression analysis was done with RT-qPCR; gene methylation analysis was done with qMSP. The significance of effects: genotype, substance and interaction genotype x substance were calculated with two-way ANOVA (SAS Enterprise Guide 8.2 update 4; SAS Institute Inc., Cary, NC, USA).

## Discussion

The main goal of the presented study was to verify the epigenetic character of gene expression stimulated by the administration of bioactive substances (prebiotic, probiotic, synbiotic) during embryo development on d 12 of egg incubation in the spleens of two different chicken genotypes.

### Meta-analysis based on published microarray data and gene selection

The effects of the *in ovo* administration of bioactive substances on broiler chickens were already proved and published elsewhere [11]. A significant, long-term effects of *in ovo* delivery of bioactive substances were also determined at the molecular level, using transcriptomic approach [10]. Gene expression silencing was determined especially in genes related to the immune responses. Delivery of synbiotic GOS with *Lactobacillus salivarius in ovo* showed a negative modulation of gene expression in the spleen [12]. Out of 50 DEG, 32 genes showed strong inhibition of mRNA expression [12]. These phenomenon of negative modulation of gene expression indicates that stimulation of the GIT microbial composition contributes to the development of feed tolerance in rapidly growing broiler chickens. As a result, metabolic energy is used for growth and development, local immune response is muted instead of maintaining the lymphatic system in excitation [31]. Maintaining active immune system is energetically costly, what might adversely affect the body growth [32]. Intestinal epithelium is constantly exposed to large amount of foodborne and bacterial antigens [33]. Gut-associated lymphoid tissue (GALT) is a vital part of the immune system that protects the body from foreign antigens and pathogens, while also tolerates commensal feed antigens. Peripheral part of immune system (spleen) is the main organ which generates an immune response. It plays a significant role in the organism protection from invading pathogens and antigens, maintaining the immune homeostasis and tissue regeneration processes [34]. GALT developed regulatory and anti-inflammatory mechanisms which eliminate or tolerate microbiota [35]. These mechanisms control host responses’ and develop tolerance to pathogens, what leads to recognition of commensal bacteria and activation of transient and non-inflammatory immune response [36].

Hereby, we present the first comprehensive study of gene expression and gene methylation levels analysis after administration of prebiotic, probiotic, as well as the synbiotic, in two extremely different genotypes of chickens. Earlier studies on which the meta-analysis was based concerned only transcriptome changes after administration of prebiotic and synbiotic in chicken broilers [10, 12].

Some differentially methylated genes have been relatively hypomethylated, suggesting that administration of bioactive substances may be associated with lower level or reduction of methylation, which consequently leads to changes in gene expression. However, the effect of methylation on gene expression also depends on many factors, such as the location of CpG, which does not allow a clear conclusion to be made regarding the increase or decrease of gene expression [37]. Little is known about the spleen DNA methylome and its potential cause-and-effect role in shaping the immune response in chicken. Due to the fact that methylation is tissue- dependent, it was necessary to select the tissue which plays a key role in the context of poultry production. It is unknown if transcriptional silencing of gene expression in chicken tissues after *in ovo* stimulation as shown in our previous experiments is associated with epigenetic mechanisms such as DNA methylation or histone acetylation. Identifying mechanisms that cause gene silencing would be key to understanding the molecular basis of environmental impact [38].

Literature provides the evidence that microbiome has a significant impact on the regulation of epigenetic mechanisms in mammals [39]. It has also been shown that there is a change in the microbiological profile in chicken intestines due to the *in ovo* administration of the GOS prebiotic [40] and the synbiotic consisting of GOS and lactic acid bacteria [11]. In this study, GOS was used as a prebiotic, lactic acid bacteria as a probiotic, and their combination in the form of a synbiotic, proving that their *in ovo* administration cause transcriptomic and epigenetic changes at the level of DNA methylation of genes in spleen. Despite the well-known intestinal microbiota and the growing knowledge of epigenetic regulation such as methylation, there are only few studies combining both issues [23, 41].

### Genotype-dependent methylation

Global methylation analysis showed differences between two distinct chicken genotypes stimulated *in ovo* at the early stage of embryo development. These analyzes were based on two genotypes of a different origin and selection history - the broiler chicken line and the native breed of dual purpose hens. The experimental conditions were exactly the same for both genotypes. Broiler chicken - Ross 308 is a meat-type which was created as a result of the ongoing genetic intensive selection program. It is characterized by high resistance to diseases, an excellent pace of weight gain, excellent production parameters, namely the growth rate and feed efficiency in large-scale production. Breeding of chicken broilers usually take place in commercial hatcheries. The post – hatching process due to chicken’s vaccination and transport cause so called hatching window, i.e. a gap in the access to feed and water. At this time, the possibility of inoculation of the microbiota is reduced, which results in microbiota development disorders [42]. Green-legged Partridge is a dual purpose Polish native breed. It is characterized by high disease resistance and low environmental and food requirements [43]. The GP was included in the genetic resources conservation program in a state where no selection was carried out. This may result in differentiation in response to the stimulation of the intestinal microbiota directly, and indirectly of the immune system, by external factors. Earlier research shows that *in ovo* administration of a synbiotic affects the development of the immune organs: bursa of Fabricius (in broiler chickens) and spleen (in dual purpose chickens). Improvement of spleen development in GP by delivery of synbiotics *in ovo* affects the sensitivity of the immune system to immunomodulating environmental factors [44]. These two genotypes (Ross and GP) are characterized by a diverse gene methylation level as a response to bioactive substances given during embryo development. It can be assumed that the difference is due to the genotype. GP is more resistant and more environmentally adapted, therefore environmental factors do not show such strong effects as in chicken broilers. GP native chickens and Ross broilers were the subject of the analysis in our previous studies. We have determined the morphological differences and immune responses to environmental antigens - lipoteichoic acid (LTA) and lipopolysaccharide (LPS) in broilers (Ross) and native chickens (GP) stimulated *in ovo* with bioactive compounds (prebiotic, probiotic, and synbiotic) [45]. *In ovo* stimulation enhanced colonization of the immune organs with lymphocytes in broilers to higher extent than in native chickens. It might be speculated that there was not much potential for further stimulation in GP. However, the significant changes in the immune system morphology expressed by the increased number of germinal centers in spleen was determined in native chickens (but not in broilers) [45]. The number of germinal centers in spleen indicates the activation of humoral immunity in animals by T-dependent antigens. Immune responses triggered in chickens stimulated *in ovo* are breed-dependent. The splenic (systemic) immune responses of broilers and native chickens challenged with LTA and LPS also showed distinct patterns. Genotype influenced gene expression signatures of all immune-related genes analyzed in spleen of broilers and native chickens (*P* < 0.001) (Slawinska, submitted). The higher number of cytokines was up-regulated in broiler chickens in comparison to native chickens. We conclude that the GP has more potent immune system than Ross, assessed by higher proportion of immune cells in spleen. GP is also less sensitive to environmental changes such as external stimulation of the microbiota, which can be indirectly observed in gene methylation levels.

### Substance-dependent methylation

Analysis of the global methylation in spleen showed statistically significant differences between the substances administered *in ovo* in Ross broiler chicken. The obtained results indicate that the response after administration of the probiotic is similar to the control. However, the prebiotic and synbiotic differ significantly from the probiotic, with no differences between the prebiotic and the synbiotic. It could be speculated that the administration of an exogenous dose of bacteria into the egg does not constitute such a strong environmental signal as the administration of a prebiotic or synbiotic. It can also be assumed that in the case of synbiotic, the prebiotic component plays a key role in modulation of methylation and expression profiles. Analysis of the expression of single genes confirmed statistically significant negative regulation of all analyzed genes after *in ovo* administration of the prebiotic and synbiotic in Ross. In some cases negative regulation of gene expression can be related to methylation level of the gene (e.g. *IKZF1* and *NR4A3* after PRE treatment in ROSS). Our analysis showed that *in ovo* administration of various substances differentiated the level of gene methylation. The perinatal period is crucial in the reprogramming of the microbiota, enabling colonization of the gastrointestinal tract of the embryo with beneficial bacteria before hatching [46]. *In ovo* stimulation performed on the d 12 of egg incubation assumes the administration of bioactive substances during embryonic development and stimulation of the native intestinal microbiota of the embryo before hatching. The prebiotic can penetrate the subcutaneous membrane and penetrate the embryonic circulatory system; while the probiotic becomes available during hatching, when the membrane is broken [42]. GOS impact on the modulation of gene expression after *in ovo* administration to chicken broilers has been extensively described [10, 40]. Relative analysis of the number of bacteria in the intestinal contents after GOS administration showed that its effect depends on the segment of the intestine. It mainly affects the number of *Bifidobacterium* spp. and *Lactobacillus* spp. [40].

## Conclusions

In summary, negative regulation of the gene expression after administration of bioactive substances *in ovo* on d 12 of egg incubation of broiler chicken and native polish chicken may have an epigenetic character. Epigenetic mechanisms depend on the genotype and the substance administered *in ovo*. Epigenetic nature of this research is a new direction of microbial reprogramming in poultry and extension of issues related to host-microbiome interaction. This study indicates that there is potential in bioactive substances administered *in ovo* to target silencing gene expression in spleen that is behind DNA methylation.

## Data Availability

The datasets used and/or analyzed during the current study are available from the corresponding author on request.

## Abbreviations

miRNA: micro RNA
mRNA: Messenger RNA
GOS: Galaktooligosaccharides
P1: GOS
P2: Inulin
S1: *Lactococcus lactis* subsp. *lactis* + inulin
S2: *Lactobacillus salivarius* + GOS
S3: *Lactococcus lactis* subsp. *cremoris* + GOS
FC: Fold change
GP: Green-legged partridge
Ross: Ross308 broiler chicken
PRO: Probiotic
PRE: Prebiotic
SYN: Synbiotic
RT-qPCR: Reverse transcription-quantitative polymerase chain reaction
qMSP: Real-time quantitative methylation-specific polymerase chain reaction
Ct: Cycle threshold
M: Methylated/average fluorescence intensity of the methylated product
U: Unmethylated/average fluorescence intensity of the unmethylated product
F: Forward
R: Reverse
SD: Standard deviation
CV: Coefficient of variation
LTA: Lipoteichoic acid
LPS: Lipopolysaccharide
ACTB: Beta actin
G6PDH: Glucose-6-phosphate dehydrogenase
CD72: CD72 molecule
CXCR5: C-X-C chemokine receptor type 5
NFATC1: Nuclear factor of activated T-cells 1
SYK: Tyrosine-protein kinase
CYR61: Cysteine-rich angiogenic inducer 61
NR4A3: Nuclear receptor subfamily 4 group A member 3
SERPING1: Serpin family G member 1
TNFRSF14: TNF receptor superfamily member 14
IKZF1: IKAROS family zinc finger 1
KLHL6: Kelch like family member 6
ANGPTL4: Angiopoietin-like 4

## References

[1] Tiffon C (2018). The impact of nutrition and environmental epigenetics on human health and disease. Int J Mol Sci.

[2] Jaenisch R, Bird A (2003). Epigenetic regulation of gene expression: how the genome integrates intrinsic and environmental signals. Nat Genet.

[3] Pan X, Chen F, Wu T, Tang H, Zhao Z (2009). Prebiotic oligosaccharides change the concentrations of short-chain fatty acids and the microbial population of mouse bowel. J Zhejiang Univ Sci B.

[4] Paul B, Barnes S, Demark-Wahnefried W, Morrow C, Salvador C, Skibola C, Tollefsbol TO (2015). Influences of diet and the gut microbiome on epigenetic modulation in cancer and other diseases. Clin Epigenetics.

[5] Ajithdoss DK, Dowd SE, Suchodolski JS (2012). Genomics of probiotic–host interactions. Direct-fed Microbials and prebiotics for animals.

[6] Alloui MN, Szczurek W, Science F (2013). The usefulness of prebiotics and probiotics in modern poultry nutrition: a review. Ann Anim Sci.

[7] Yang Y, Iji PA, Choct M (2009). Dietary modulation of gut microflora in broiler chickens: a review of the role of six kinds of alternatives to in-feed antibiotics. Worlds Poult Sci J.

[8] Sansonetti PJ, Di Santo JP (2007). Debugging how bacteria manipulate the immune response. Immunity..

[9] Pilarski R, Bednarczyk M, Lisowski M, Rutkowski A, Bernacki Z, Wardeńska M (2005). Assessment of the effect of alpha-galactosides injected during embryogenesis on selected chicken traits. Folia Biol (Cracow).

[10] Slawinska A, Plowiec A, Siwek M, Jaroszewski M, Bednarczyk M (2016). Long-term transcriptomic effects of prebiotics and synbiotics delivered in ovo in broiler chickens. Plos One.

[11] Dunislawska A, Slawinska A, Stadnicka K, Bednarczyk M, Gulewicz P, Jozefiak D, Bednarczyk M, Siwek M (2017). Synbiotics for broiler chickens—in vitro design and evaluation of the influence on host and selected microbiota populations following in ovo delivery. Plos One.

[12] Dunislawska A, Slawinska A, Bednarczyk M, Siwek M (2019). Transcriptome modulation by in ovo delivered Lactobacillus synbiotics in a range of chicken tissues. Gene..

[13] Shen L, Waterland RA (2007). Methods of DNA methylation analysis. Curr Opin Clin Nutr Metab Care.

[14] Hargan-Calvopina J, Taylor S, Cook H, Hu Z, Lee SA, Yen MR, Chiang YS, Chen PY, Clark AT (2016). Stage-specific Demethylation in primordial germ cells safeguards against precocious differentiation. Dev Cell.

[15] Gao F, Niu Y, Sun YE, Lu H, Chen Y, Li S, Kang Y, Luo Y, Si C, Yu J, Li C, Sun N, Si W, Wang H, Ji W, Tan T (2017). De novo DNA methylation during monkey pre-implantation embryogenesis. Cell Res.

[16] Smith ZD, Meissner A (2013). DNA methylation: roles in mammalian development. Nat Rev Genet.

[17] Greenberg MVC, Bourc’his D (2019). The diverse roles of DNA methylation in mammalian development and disease. Nat Rev Mol Cell Biol.

[18] Kvist J, Goncalves Anthanasio C, Solari OS, Brown J, Colbourne J, Pfrender M (2018). Pattern of DNA methylation in Daphnia: evolutionary perspective. Genome Biol Evol.

[19] Li E, Zhang Y (2014). DNA methylation in mammals. Cold Spring Harb Perspect Biol.

[20] Szklarczyk D, Franceschini A, Kuhn M, Simonovic M, Roth A, Minguez P, et al. The STRING database in 2011: Functional interaction networks of proteins, globally integrated and scored. Nucleic Acids Res. 2011;39 suppl. 1(Database issue):D561–8. 10.1093/nar/gkq973 PMC3013807 21045058

[21] Bednarczyk M, Stadnicka K, Kozłowska I, Abiuso C, Tavaniello S, Dankowiakowska A, Sławińska A, Maiorano G (2016). Influence of different prebiotics and mode of their administration on broiler chicken performance. Animal..

[22] Maniatis T, Fritsch E, Sambrook J. Molecular cloning: a laboratory manual. Cold Spring Harbor Laboratory. In: Cold Spring Harbor Laboratory, Cold Spring Harbor, NY. 1982.

[23] Dunislawska A, Slawinska A, Siwek M (2020). Hepatic DNA methylation in response to early stimulation of microbiota with Lactobacillus synbiotics in broiler chickens. Genes (Basel).

[24] Gryzinska M, Blaszczak E, Strachecka A, Jezewska-Witkowska G (2013). Analysis of age-related global DNA methylation in chicken. Biochem Genet.

[25] Ye J, Coulouris G, Zaretskaya I, Cutcutache I, Rozen S, Madden TL (2012). Primer-BLAST: a tool to design target-specific primers for polymerase chain reaction. BMC Bioinformatics.

[26] Livak KJ, Schmittgen TD (2001). Analysis of relative gene expression data using real-time quantitative PCR and the 2(−Delta Delta C(T)) method. Methods..

[27] Sevane N, Bialade F, Velasco S, Rebolé A, Rodríguez ML, Ortiz LT, Cañón J, Dunner S (2014). Dietary inulin supplementation modifies significantly the liver transcriptomic profile of broiler chickens. Plos One.

[28] De Boever S, Vangestel C, De Backer P, Croubels S, Sys SU (2008). Identification and validation of housekeeping genes as internal control for gene expression in an intravenous LPS inflammation model in chickens. Vet Immunol Immunopathol.

[29] Vandesompele J, De Preter K, Pattyn F, Poppe B, Van Roy N, De Paepe A (2002). Accurate normalization of real-time quantitative RT-PCR data by geometric averaging of multiple internal control genes. Genome Biol.

[30] Fackler MJ, McVeigh M, Mehrotra J, Blum MA, Lange J, Lapides A, Garrett E, Argani P, Sukumar S (2004). Quantitative multiplex methylation-specific PCR assay for the detection of promoter hypermethylation in multiple genes in breast cancer. Cancer Res.

[31] Kominsky DJ, Campbell EL, Colgan SP (2010). Metabolic shifts in immunity and inflammation. J Immunol.

[32] Van Der Most PJ, De Jong B, Parmentier HK, Verhulst S (2011). Trade-off between growth and immune function: a meta-analysis of selection experiments. Funct Ecol.

[33] Ghadimi D, Helwig U, Schrezenmeir J, Heller KJ, de Vrese M (2012). Epigenetic imprinting by commensal probiotics inhibits the IL-23/IL-17 axis in an in vitro model of the intestinal mucosal immune system. J Leukoc Biol.

[34] Lewis SM, Williams A, Eisenbarth SC (2019). Structure and function of the immune system in the spleen. Sci Immunol.

[35] Brisbin JT, Zhou H, Gong J, Sabour P, Akbari MR, Haghighi HR, Yu H, Clarke A, Sarson AJ, Sharif S (2008). Gene expression profiling of chicken lymphoid cells after treatment with Lactobacillus acidophilus cellular components. Dev Comp Immunol.

[36] Galdeano CM, de Moreno de LeBlanc A, Vinderola G, Bonet MEB, Perdigón G (2007). Proposed model: mechanisms of immunomodulation induced by probiotic bacteria. Clin Vaccine Immunol.

[37] Moore LD, Le T, Fan G (2013). DNA methylation and its basic function. Neuropsychopharmacol..

[38] Ghavifekr Fakhr M, Farshdousti Hagh M, Shanehbandi D, Baradaran B (2013). DNA methylation pattern as important epigenetic criterion in cancer. Genet Res Int..

[39] Ramos-Molina B, Sánchez-Alcoholado L, Cabrera-Mulero A, Lopez-Dominguez R, Carmona-Saez P, Garcia-Fuentes E, et al. Gut microbiota composition is associated with the global DNA methylation pattern in obesity. Front Genet. 2019;10. 10.3389/fgene.2019.00613. 10.3389/fgene.2019.00613 PMC6616130 31333715

[40] Slawinska A, Dunislawska A, Plowiec A, Radomska M, Lachmanska J, Siwek M, Tavaniello S, Maiorano G (2019). Modulation of microbial communities and mucosal gene expression in chicken intestines after galactooligosaccharides delivery in Ovo. Plos One.

[41] Berghof TVL, Parmentier HK, Lammers A (2013). Transgenerational epigenetic effects on innate immunity in broilers: an underestimated field to be explored. Poult Sci.

[42] Siwek M, Slawinska A, Stadnicka K, Bogucka J, Dunislawska A, Bednarczyk M (2018). Prebiotics and synbiotics – in ovo delivery for improved lifespan condition in chicken. BMC Vet Res.

[43] Siwek M, Wragg D, Sławińska A, Malek M, Hanotte O, Mwacharo JM (2013). Insights into the genetic history of green-legged Partridgelike fowl: MtDNA and genome-wide SNP analysis. Anim Genet.

[44] Sławińska A, Siwek M, Zylińska J, Bardowski J, Brzezińska J, Gulewicz KA (2014). Influence of synbiotics delivered in ovo on immune organs development and structure. Folia Biol (Praha).

[45] Madej JP, Skonieczna J, Siwek M, Kowalczyk A, Łukaszewicz E, Slawinska A (2020). Genotype-dependent development of cellular and humoral immunity in spleen and cecal tonsils of chickens stimulated in ovo with bioactive compounds. Poult Sci.

[46] Roto SM, Kwon YM, Ricke SC (2016). Applications of in ovo technique for the optimal development of the gastrointestinal tract and the potential influence on the establishment of its microbiome in poultry. Front Vet Sci.

